# β-carotene Rescues Busulfan Disrupted Spermatogenesis Through Elevation in Testicular Antioxidant Capability

**DOI:** 10.3389/fphar.2021.593953

**Published:** 2021-02-15

**Authors:** Dongxue Ma, Pengfei Han, Mingji Song, Hongfu Zhang, Wei Shen, Guian Huang, Minghui Zhao, Qingyuan Sun, Yong Zhao, Lingjiang Min

**Affiliations:** ^1^ College of Animal Sciences and Technology, Qingdao Agricultural University, Qingdao, China; ^2^ College of Life Sciences, Qingdao Agricultural University, Qingdao, China; ^3^ State Key Laboratory of Animal Nutrition, Institute of Animal Sciences, Chinese Academy of Agricultural Sciences, Beijing, China

**Keywords:** β-carotene, spermatogenesis, antioxidant, improvement, reactive oxygen species

## Abstract

β-carotene, precursor of vitamin A, is an excellent antioxidant with many beneficial properties. It is a lipid-soluble antioxidant and a very effective quencher of reactive oxygen species (ROS) to reduce the oxidative stress. In contrast to vitamin A, β-carotene is not toxic even consumed in higher amount when it is delivered from natural plant products. Recently, we found that β-carotene acts as a potential antioxidant in the oocyte to improve its quality. Even though many studies have been reported that β-carotene has the beneficial contribution to the ovarian development and steroidogenesis, it is unknown the effects of β-carotene on the spermatogenesis. This investigation aimed to explore the hypothesis that β-carotene could improve spermatogenesis and the underlying mechanism. And we found that β-carotene rescued busulfan disrupted spermatogenesis in mouse with the increase in the sperm concentration and motility. β-carotene improved the expression of genes/proteins important for spermatogenesis, such as VASA, DAZL, SYCP3, PGK2. Moreover, β-carotene elevated the testicular antioxidant capability by the elevation of the antioxidant glutathione and antioxidant enzymes SOD, GPX1, catalase levels. In conclusion, β-carotene may be applied for the infertile couples by the improvement of spermatogenesis, since, worldly many couples are infertile due to the idiopathic failed gametogenesis (spermatogenesis).

## Introduction

Infertility is a growing issue worldwide which now impacts 10%–15% of all couples with half of the cases being attributed to male factors (Zhou et al., 2016; Wang et al., 2018). Epidemiological studies reported that spermatozoa concentration was diminished, worldwide, by over 50% from 1973 to 2011, at the same time sperm motility was also decreased dramatically (Centola et al., 2016; Levine et al., 2017). Various factors are involved in the rapid decline of semen quality, such as environmental toxins (Checa Vizcaíno et al., 2016; Skakkebaek et al., 2016; Virtanen et al., 2017; Han et al., 2019; Zhang et al., 2019), high fat diet (Ding et al., 2020), and cancer treatments (Trost and Brannigan 2012; Vakalopoulos et al., 2015). Many investigations have attempted to improve spermatogenesis and male fertility using a busulfan treated animal model. Busulfan can cause azoospermia by destroying testicular germ cells and increasing sperm abnormalities (Chi et al., 2013; Jung et al., 2015; Liu et al., 2019). It has been reported that molybdenum (Mo) rescued mouse spermatogenesis by the improvement of male germ cell development s, and maintaining of blood hormone levels (testosterone, estradiol, and luteinizing hormone) (Liu et al., 2019). Olive leaf extract has been found to improve busulfan disrupted spermatogenesis (Ganjalikhan Hakemi et al., 2019). Korean red ginseng can rescue busulfan induced the disruption of spermatogenesis (Jung et al., 2015). Genistein has been reported to decrease intra-testicular testosterone (ITT) level and to rescue busulfan disrupted spermatogenesis in rats (Chi et al., 2013). Furthermore, Zhao et al. (2020) found that alginate oligosaccharides (AOS) improved busulfan disrupted spermatogenesis at the single cell level.

β-carotene (C_40_H_56_) is one of the major dietary carotenoids (Johnson 2002). β-carotene, alpha-carotene and beta cryptoxanthin are the major precursors of vitamin A. Vitamin A has been reported to pose a number of health advantages such as retainment of eye health, epithelial function, embryonic development, immune system function, and the cardiovascular function, and even the reduction of cancer incidence (Boon et al., 2010). β-carotene, widely present in vegetables and fruits such as carrot, mango, and spinach, is the precursor of vitamin A (Boon et al., 2010; Pysz et al., 2016). It is an effective lipid-soluble antioxidant to maintain the reducing micro-environment in biological systems (Gu et al., 2017; Nishino et al., 2017). Consumption of high level of vitamin A can result in toxicity, while β-carotene from natural plant products is nontoxic even consumed in large amount. Moreover, β-carotene from fruits and vegetables not only improves nutritional status of vitamin A but also prevents the incidence of cancer (Fiedor and Burda, 2014). Daily supplementation of β-carotene significantly increases β-carotene and retinol bioavailability, consequently to benefit follicular development and oocyte maturation (De Bie et al., 2016). Short-term dietary β-carotene supplement ameliorates ovarian steroidogenesis with the increase in the production of progesterone (Arellano-Rodriguez et al., 2009), and benefits serum insulin concentrations in goats (Meza-Herrera et al., 2013). We found that β-carotene acts as a potential antioxidant in the oocyte to improve its quality (Yu et al., 2019). Moreover, β-carotene has been found to act as a valuable protective agent to improve heat stress or titanium oxide nanoparticle induced spermatogenic disorders due to its potent antioxidative effects (Orazizadeh et al., 2014; Lin et al., 2016). Even though early studies have suggested that β-carotene poses the beneficial contribution to the ovarian development and steroidogenesis, and spermatogenesis at specific condition, it is not understood the effects of β-carotene on the spermatogenesis especially on the conditional azoospermia (starting from the pubertal window). The purpose of this investigation was to explore the hypothesis that β-carotene could improve spermatogenesis at the pubertal window, and the underlying mechanism.

## Materials and Methods

### Study Design

This study was approved by the Committee on the Ethics of Animal Experiments of Qingdao Agricultural University IACUC (Institutional Animal Care and Use Committee; Approval #: QAUIACUC20191102M) in strict accordance with the recommendations in the Guide for the Care and Use of Laboratory Animals of the National Institutes of Health (Zhang et al., 2018; Zhao et al., 2020). (Detailed Materials and Methods in the Supplementary Material) Mice were maintained under a light: dark cycle of 12:12 h and at a temperature of 23°C and humidity of 50%–70%, and with free access to food (chow diet) and water (Zhang et al., 2018; Zhao et al., 2020). The main purpose of this investigation was to explore the rescuing effects of β-carotene on spermatozoa quality and the underlying mechanisms.

Three-week-old ICR male mice were given a single injection of busulfan [40 mg/kg body weight (BW)] (Jung et al., 2015). We would like to explore the beneficial advantages of β-carotene on the spermatogenesis at pubertal period (Dutta and Sengupta 2016). The following day, the mice were dosed with corn oil as the control or β-carotene in corn oil via oral gavage (0.1 ml/mouse/d). β-carotene dosing solution was freshly prepared on a daily basis and delivered every morning for five weeks. There were fourteen treatment groups (30 mice/treatment): 1) Control (vehicle control, corn oil); 2) β-caro 1 (β-carotene 1 mg/kg BW); 3) β-caro 10 (β-carotene 10 mg/kg BW); 4) β-caro 50 (β-carotene 50 mg/kg BW); 5) β-caro 100 (β-carotene 100 mg/kg BW); 6) β-caro 200 (β-carotene 200 mg/kg BW); 7) β-caro 300 β-carotene 300 mg/kg BW); 8) B0 (busulfan alone); 9) B + β-caro 1 (busulfan plus β-carotene 1 mg/kg BW); 10) B + β-caro 10 (busulfan plus β-carotene 10 mg/kg BW); 11) B + β-caro 50 (busulfan plus β-carotene 50 mg/kg BW); 12) B + β-caro 100 (busulfan plus β-carotene 100 mg/kg BW); 13) B + β-caro 200 (busulfan plus β-carotene 200 mg/kg BW); 14) B + β-caro 300 (busulfan plus β-carotene 300 mg/kg BW). After treatment, the mice were humanely euthanized to collect samples for different analyses.

### Spermatozoa Motility Determined by a Computer-Assisted Sperm Analysis System

Spermatozoa motility and concentration were assessed by a computer-assisted sperm assay (CASA) method according to World Health Organization guidelines (WHO, 2010; Zhao et al., 2016). The spermatozoa motility data were present grade A + grade B (WHO, 2010).

### Measurement of Plasma Steroid Hormones

Plasma testosterone (T) and oestrogen (E) levels were quantified by mouse testosterone Elisa Kit and mouse estradiol Elisa Kit, respectively, from Nanjing Jiancheng Bioengineering Institute (Nanjing, China) as reported in our early publication (Wang et al., 2016).

### Measurement of Plasma AST and ALT

Plasma aspartate aminotransferase (AST; Cat #: C010-2-1) and alanine aminotransferase (ALT; Cat #: C009-2-1) were measured by the kits from Nanjing Jiancheng Bioengineering Institute (Nanjing, China) as reported in our previous publication (Wang et al., 2016).

### Measurement of Testis Antioxidant Enzyme Activity

The activity of catalase (Cat #: A007-1-1), GPX1 (Cat #: A005), SOD (Cat #: A001-3), T-AOC (Cat #: A015-2-1) in mouse testis were detected by the kits from Nanjing Jiancheng Bioengineering Institute (Nanjing, China) as reported in our previous publication (Wang et al., 2016).

### Measurement of Testis Glutathione/GSSG

The testis levels of GSH/GSSG (Cat #: S0053) were detected by the kits from Beyotime Bioengineering Institute (Shanghai, China) as reported in our previous publication (Wang et al., 2016).

### Western Blotting

Western blotting analysis was followed the procedure reported in our previous publications (Wang et al., 2016; Zhao et al., 2016; Zhang et al., 2018). The sources of primary antibodies are listed in Supplementary Table S1. The experiments were repeated >3 times. The intensity of WB bands was determined by image J software.

### Immunofluorescent Staining

The procedure for immunofluorescent staining was reported in our recent publications (Wang et al., 2016; Zhao et al., 2016; Zhang et al., 2018). Supplementary Table S1 lists the primary antibodies. Testis sections (5 μm) were prepared and subjected to antigen retrieval and immunostaining as previously described (Zhang et al., 2018). Briefly, sections were first blocked with normal goat serum in PBS, followed by incubation (1:150 in PBS-1% BSA) with primary antibodies at 4°C overnight. After a brief wash, sections were incubated with an Alexa 546-labeled goat anti-rabbit or donkey anti-goat secondary Abs (1:100 in PBS; Beyotime Institute of Biotechnology, Shanghai, China) at room temperature for 30 min and then counterstained with 4′,6-diamidino-2-phenylindole (DAPI). The stained sections were visualized using a Nikon Eclipse TE2000-U fluorescence microscope (Nikon, Inc., Melville, NY), and the captured fluorescent images were analyzed using MetaMorph software. A minimum of 1,000 cells were counted for each section, and a minimum of two to three tissue sections per animal were analyzed. Three animals from the control or treatment groups were analyzed. The number of positive cells was expressed as the percentage of total cells counted. The data was expressed as the average ±SD, N > 3.

### Statistical Analysis

The data were determined by SPSS statistical software (IBM Co., NY) with one-way analysis of variance (ANOVA) following by LSD multiple comparison test. All groups were compared with each other for every parameter. The data were shown as the mean ± SEM. Statistically significant was based on *p* < 0.05.

## Results

### β-carotene Increased the Motility and Concentration of Spermatozoa After Busulfan Treatment

**FIGURE 1:**
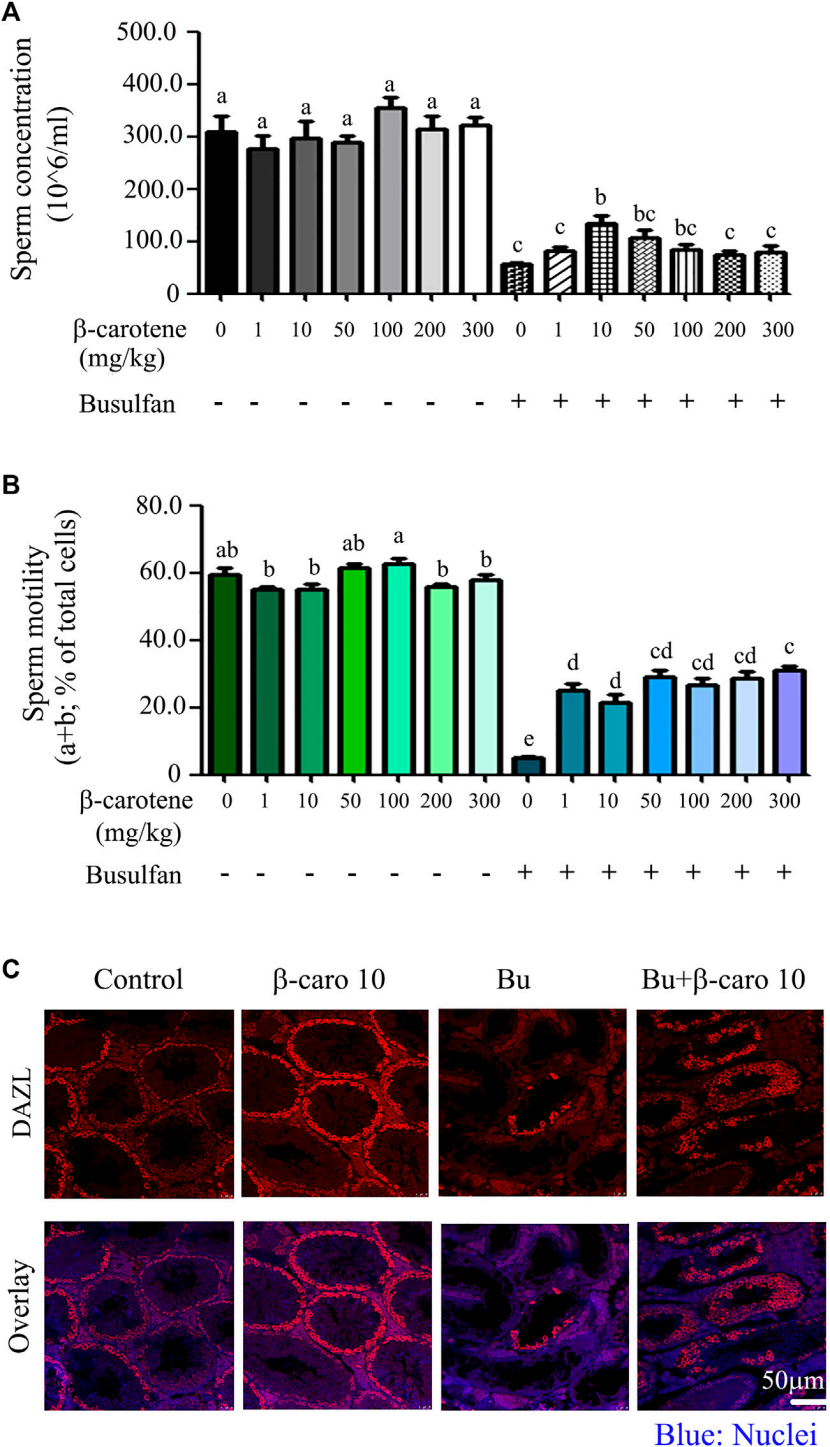
Mouse sperm motility, concentration, and DAZL expression. **(A)** Mouse sperm concentration. The y-axis represents the concentration. The x-axis represents the treatment (n = 30/group). ^a,b,c^ Means not sharing a common superscript are different (*p <* 0.05). **(B)** Mouse sperm motility. The y-axis represents the percentage of cells. The X-axis represents the treatment (n = 30/group). ^a,b,c^ Means not sharing a common superscript are different (*p <* 0.05). **(C)** IHF for germ cell marker DAZL.

As shown in Figures 1A,B, β-carotene at the concentration of 1-300 mg/kg body weight (BW) did not affect mouse sperm concentration or motility so much. However, after busulfan treatment, β-carotene significantly increased sperm concentration at 10 mg/kg BW compared to busulfan alone (Figure 1A). 50 and 100 mg/kg β-carotene did not significantly increased mouse sperm concentration compared to busulfan alone, which may be due to the dose dependent effect (the better dose is around 10 mg/kg). After busulfan treatment, all six doses of β-carotene (1–300 mg/kg) significantly elevated mouse sperm motility at similar level compared to busulfan alone (Figure 1B). Because 10 mg/kg BW β-carotene produced the relatively confound effect on improving mouse sperm quality, most of the following experiments were done with this dose. Moreover, the data were confirmed by the IHF staining with the germ cell marker DAZL. Busulfan disrupted the spermatogenesis by the decrease in the germ cells, while β-carotene dramatically increased the number of germ cells than busulfan alone. The data here suggested that β-carotene can improve spermatogenesis. β-carotene did not affect body weight, organ index, and other body parameters much (Table 1). β-carotene did not improve the testis index even though it improved busulfan disrupted spermatogenesis. This may be due to β-carotene partially improved the spermatogenesis not to that degree to affect testis wet weight.

**TABLE 1:** Mouse body and plasma parameters. Data present as Average ±SEM.^a,b,c^ indicate a significant difference among different treatments (n = 20; *p* < 0.05).

	Control	Carotene 1	Carotene 10	Carotene 50
Body weight (g)	32.44 ± 2.25	36.54 ± 1.00	36.37 ± 1.38	35.34 ± 0.82
Testis index (% of body weight)	0.84 ± 0.074	0.67 ± 0.029	0.73 ± 0.027	0.72 ± 0.023
Kidney index (% of body weight)	1.94 ± 0.167	1.71 ± 0.082	1.70 ± 0.034	1.70 ± 0.059
Spleen index (% of body weight)	0.42 ± 0.05	0.36 ± 0.02	0.31 ± 0.02	0.33 ± 0.02
Liver index (% of body weight)	6.33 ± 0.52^a^	5.34 ± 0.22 ^bc^	5.50 ± 0.12 ^bc^	5.62 ± 0.22^b^
E2 (ng/L)	251.84 ± 8.87	291.75 ± 29.99	291.75 ± 17.52	286.68 ± 13.52
Testosterone (ng/L)	47.64 ± 3.69	30.40 ± 5.42	33.20 ± 6.20	37.48 ± 9.58
ALT	35.77 ± 0.77	34.83 ± 1.60	35.46 ± 0.82	34.37 ± 0.71
AST	70.6 ± 3.7	55.1 ± 1.6	66.4 ± 2.94	65.7 ± 3.0
	Busulfan	B + carotene 1	B + carotene 10	B + carotene 50
Body weight (g)	32.50 ± 0.55	32.86 ± 0.79	33.82 ± 0.56	35.73 ± 0.75
Testis index (% of body weight)	0.24 ± 0.009	0.21 ± 0.006	0.21 ± 0.012	0.19 ± 0.011
Kidney index (% of body weight)	1.72 ± 0.029	1.75 ± 0.063	1.65 ± 0.067	1.62 ± 0.048
Spleen index (% of body weight)	0.39 ± 0.016	0.38 ± 0.014	0.39 ± 0.019	0.31 ± 0.011
Liver index (% of body weight)	5.00 ± 0.098	4.80 ± 0.085	4.85 ± 0.105	5.62 ± 0.122
E2 (ng/L)	329.13 ± 29.15	273.31 ± 29.99	378.34 ± 21.88	422.15 ± 21.19
Testosterone (ng/L)	43.92.±7.29	33.92 ± 9.78	39.84 ± 6.12	40.40 ± 8.66
ALT	37.38 ± 1.13	38.71 ± 0.50	35.45 ± 1.08	38.80 ± 1.53
AST	78.6 ± 3.72	84.36 ± 7.91	75.56 ± 4.98	80.53 ± 8.87

### β-carotene Rescued Busulfan Disrupted Meiosis Process During the Spermatogenesis

**FIGURE 2:**
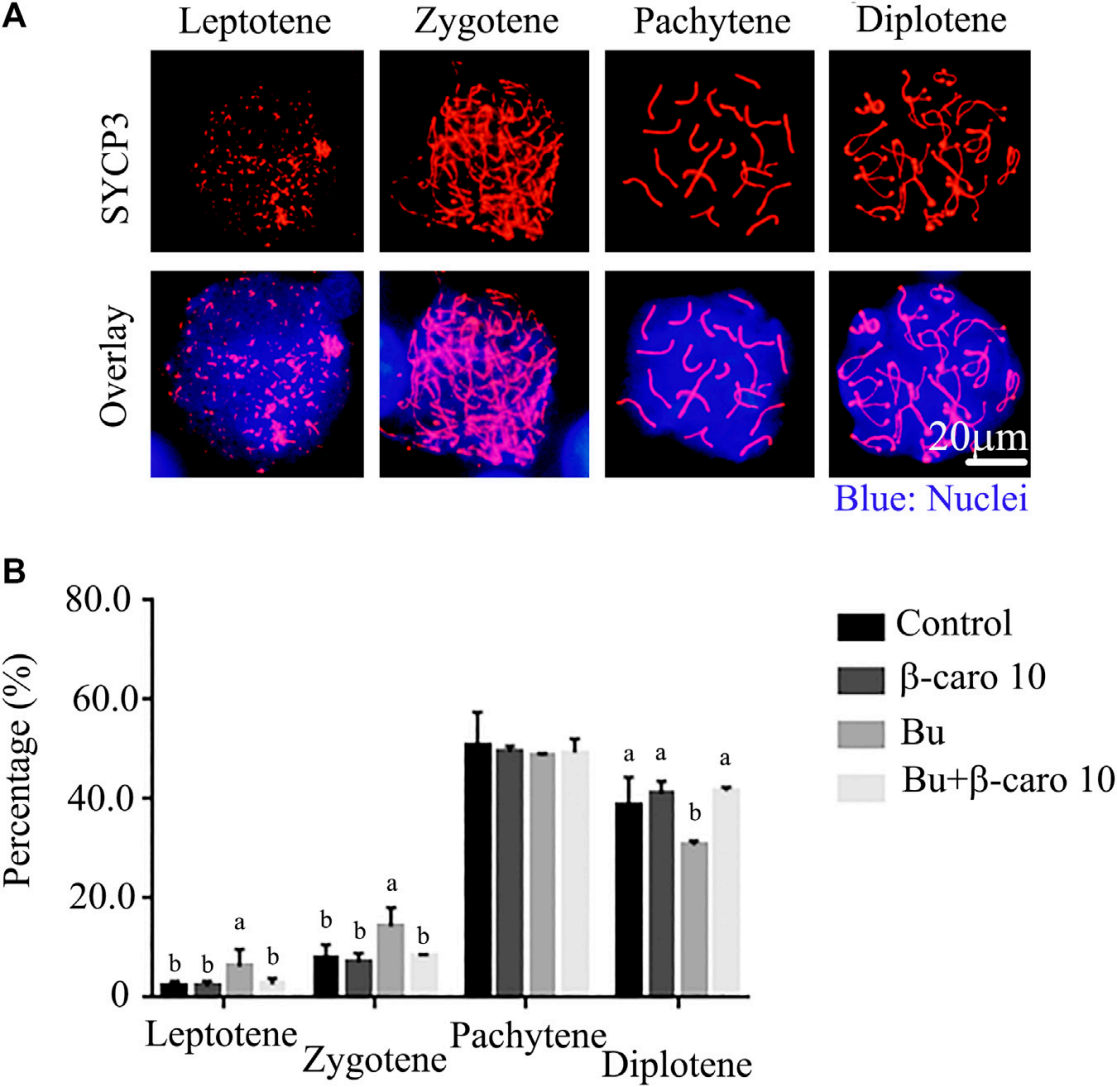
β-carotene rescued the meiosis process in spermatogenesis after busulfan treatment. **(A)** The representative images for the immunostaining of meiosis marker SYCP3 at different meiosis stages. **(B)** The percentage of cells at different meiosis stages in different treaments n = 10.

Meiosis plays important roles in the controlling germ cell development to haploid and maintaining genetic diversity. It has been reported that the abnormal meiosis will induce aneuploidy formation, even cause infertility, miscarriage or even birth defect. In current investigation, busulfan disrupted the meiosis process during the spermatogenesis with the alteration in the percentage of cells at different stages (Figure 2). The percentage of cells at leptotene and zygotene was increased while the percentage of cells at diplotene was decreased in busulfan treated mice (Figure 2B). However, β-carotene rescued busulfan disrupted meiosis process with the restoration of the percentage of the cells as similar levels in control mice at all four stage of meiosis (Leptotene, zygotene, Pachytene, and leptotene; Figure 2B).

### β-carotene Improved the Expression of Important Genes for Spermatogenesis

**FIGURE 3:**
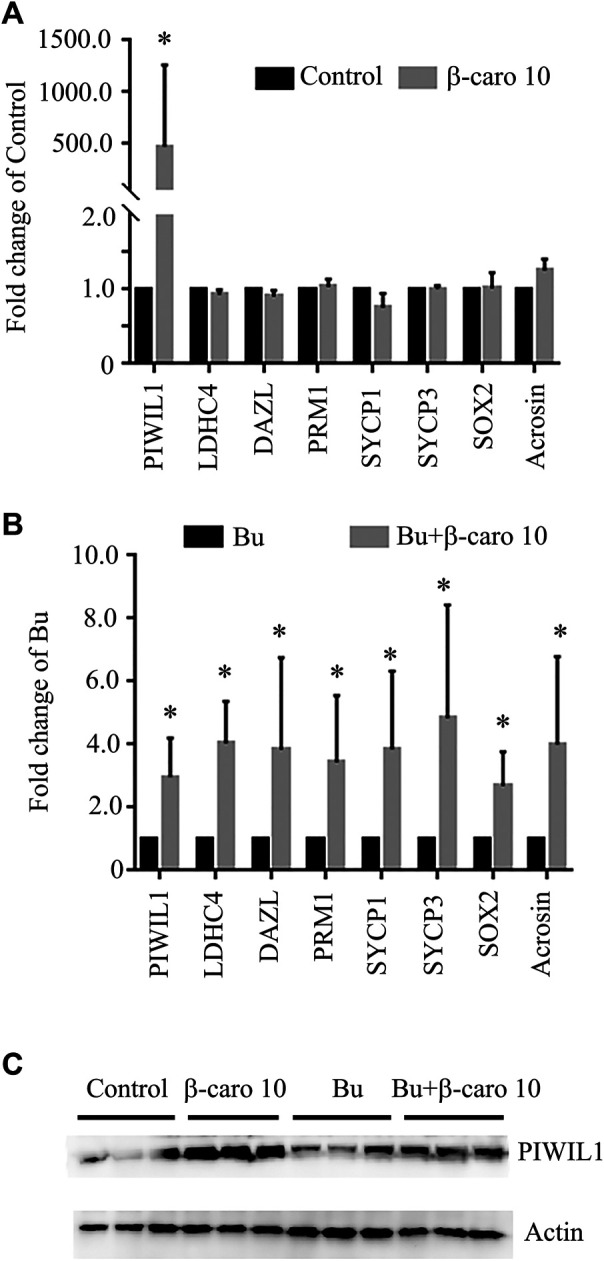
β-carotene improved the gene expression of important makers in spermatogenesis. **(A)** The expression of eight important genes in control and β-carotene groups. **(B)** The expression of eight important genes in busulfan and busulfan + β-carotene groups. **(C)** Protein expression of piwil1 in testicular samples by WB. *, *p <* 0.05.

**FIGURE 4:**
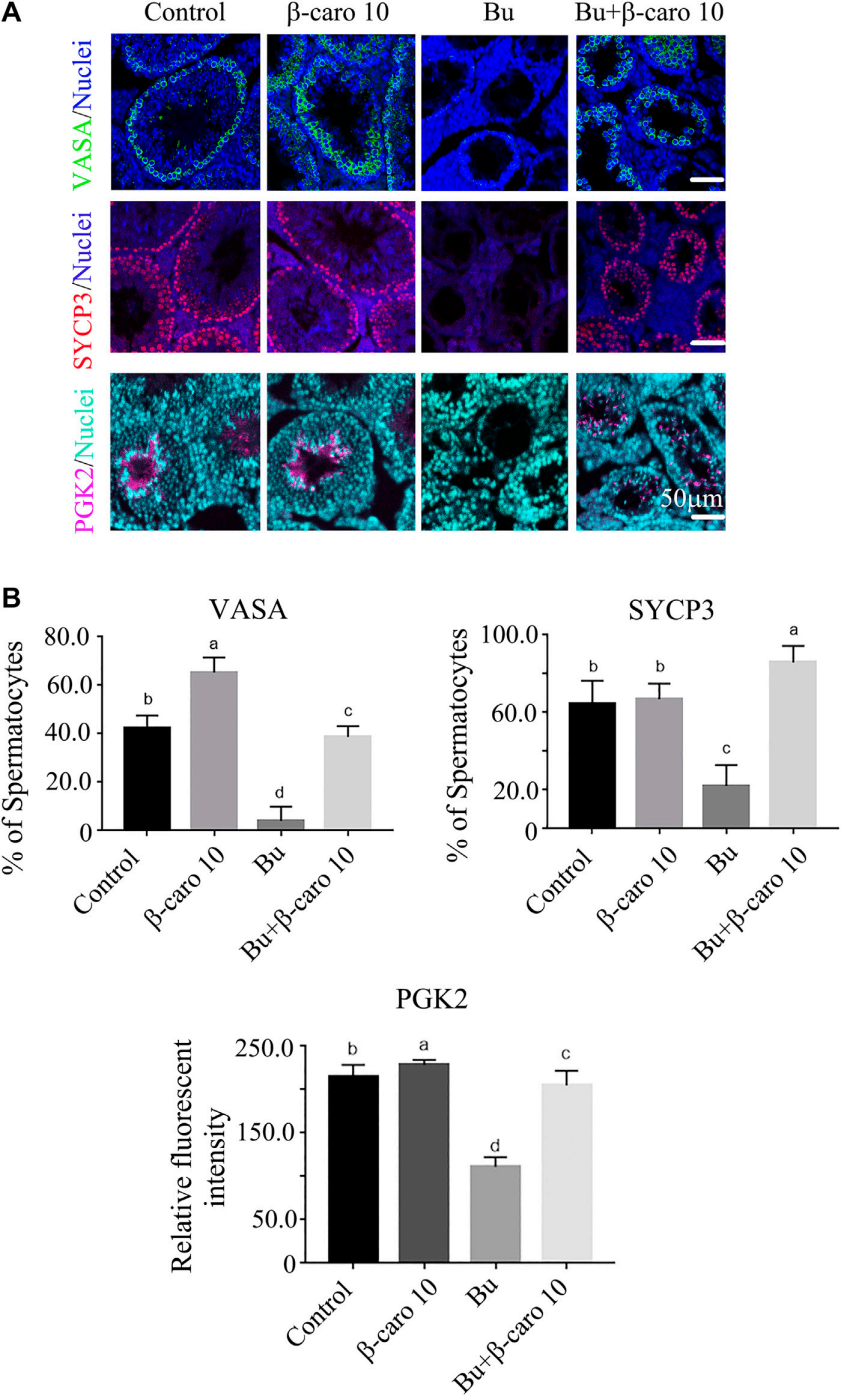
β-carotene improved the protein expression of important makers in spermatogenesis. **(A)** IHF for important genes for spermatogenesis VASA, SYCP3, and PGK2. **(B)** Quantitative data for IHF of VASA. **(C)** Quantitative data for IHF of SYCP3. **(D)** Quantitative data for IHF of PGK2. ^a,b,c,d^ Means not sharing a common superscript are different (*p <* 0.05).

In order to confirm β-carotene improving spermatogenesis and search for the mode of action of it in rescuing spermatogenesis, the gene and protein expression of important factors in spermatogenesis was determined. We did Q-RT-PCR for a lot of genes important for spermatogenesis, and we found that the expression of eight genes was increased by B + β-caro 10 group compared to Busulfan alone, while just PIWIL1 was dramatically increased by β-caro 10 compared to control (Figures 3A,B). At the same time the protein level of PIWIL1 was determined by WB. β-caro 10 significantly increased the protein level of PIWIL1 compared to control group. B + β-caro 10 significantly increase PIWIL1 protein levels compared to B alone (Figure 3C). The data here confirmed the Q-RT-PCR data. Meaning while, the protein levels of germ cell marker VASA, meiosis protein SYCP3, and sperm cell protein PGK2 were also detected by IHF. β-caro 10 significantly elevated the protein level of VASA compared to control, while busulfan significantly decreased the protein level of VASA. However, B + β-caro 10 significantly restored the protein of VASA to control level (Figures 4A,B). β-caro 10 did not affect the protein levels of SYCP3 and PGK2, however, busulfan significantly diminished these two proteins. B + β-caro 10 significantly increased these two proteins to the control levels (Figures 4A,B). The data here confirmed the Q-RT-PCR data for these important genes in spermatogenesis.

### β-carotene Improved the Anti-Oxidant Status in Testes to Rescue the Spermatogenesis

**FIGURE 5:**
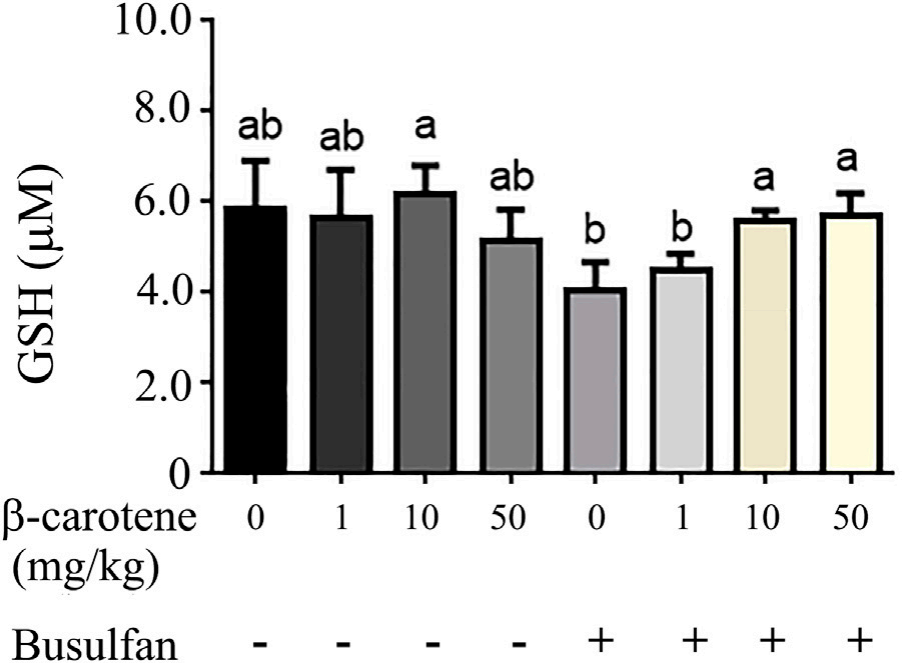
Testicular GSH level. **(A)** Busulfan + β-carotene increased the testicular GSH level compared to busulfan alone. ^a,b^ Means not sharing a common superscript are different (*p <* 0.05).

**FIGURE 6:**
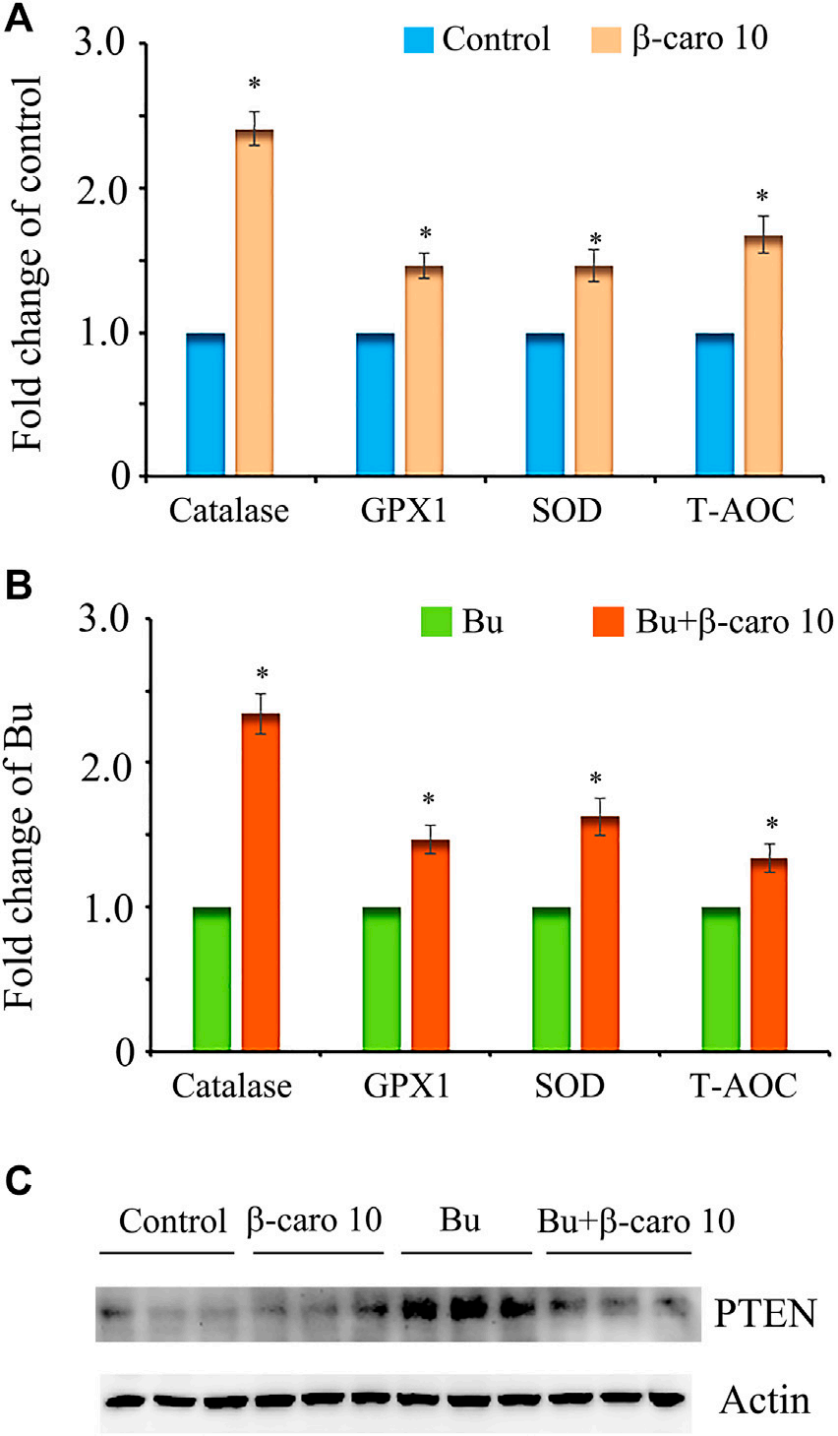
Testicular levels of the antioxidant enzymes SOD, catalase, and GPX; and T-AOC. PTEN protein level in testis samples. **(A)** Testicular levels of SOD, catalase, GPX, and T-AOC in control and β-carotene treatment groups; **(B)** Testicular levels of SOD, catalase, GPX, and T-AOC in busulfan and busulfan + β-carotene treatment groups; **(C)** Protein level of PTEN in testis samples by WB. *, *p <* 0.05.

To explore the deep mechanisms of β-carotene improving spermatogenesis, the anti-oxidant enzymes and glutathione (GSH) level in testes were determined. We and other groups have found that β-carotene can increase the anti-oxidant capability to benefit the function of the biological systems (Yu et al., 2019). GSH is an important antioxidant molecule. β-caro 10 can increase the level of testicular GSH compared to control although not significantly. Busulfan significantly decreased testicular GSH level, while B + β-caro 10 and B + β-caro 50 mg/kg groups significantly increased the testicular GSH levels compared to busulfan alone (Figure 5). At the same time, the anti-oxidant enzyme activity of catalase, GPX1 and SOD, and the total antioxidant capability (T-AOC) were determined in the testis samples. Compared to control, the activity levels of catalase, GPX1, SOD and the level of T-AOC were significantly increased by β-caro 10 mg/kg. Moreover, B+ β-caro 10 significantly elevated the activity levels of catalase, GPX1 and SOD and the level of T-AOC compared to B alone (Figures 6A,B). Furthermore, the protein level of PTEN was detected by WB. It was found that B alone increased the protein level of PTEN, while B + β-caro 10 restored the protein level of PTEN to the control level. All the data here further suggested that β-carotene can improve the antioxidant capability in testes to improve the spermatogenesis process.

## Discussion

It has been reported that the carotenoids in fruits play very important roles to prevent diseases and maintain body functions. Moreover, many investigations suggested β-carotene, lycopene, lutein, zeaxanthin and other carotenoids possess lots of health benefits. The blood concentration of β-carotene is about 2.54–3.3 μg/ml. It has been applied in many animal investigations such as cow, goat, rabbit, and sow (Arellano-Rodriguez et al., 2009; Aksak Karamese et al., 2015; Oliveira et al., 2015; Szczubiał 2015; Merhan et al., 2016). In current investigation, 1∼300 mg/kg β-carotene was used with or without busulfan for dosing mice, we found that busulfan (single dose 40 mg/kg BW at 3 weeks of age) resulted in azoospermia in mice during adulthood (8 weeks of age). The result was consistent with lots of previous reports (Chi et al., 2013; Jung et al., 2015; Liu et al., 2019). Moreover, β-carotene increased sperm concentration and motility after busulfan treatment. The data suggested that β-carotene can rescue spermatogenesis. Then, we set out to investigate the underlying mechanisms by which β-carotene rescued spermatogenesis.

β-carotene improved busulfan disrupted meiosis process. Busulfan decreased the percentage of cells in late phases, while β-carotene restored the percentage of these cells to the control level. Meiosis is an important process to keep germ cell development. And the abnormal meiosis may upset the haploid formation which may lead to the infertility, miscarriage, and so on (Men et al., 2019). Busulfan disrupted the expression of genes/proteins important for spermatogenesis, while β-carotene restored the expression of these genes/proteins to the control level.

It has been reported busulfan increased intracellular oxidative stress (Li et al., 2018). And β-carotene has been reported to be a very good antioxidant to quench singlet oxygen and free radicals which can damage cellular DNA and lipids (Haila et al., 1997; Sies and Stahl 1998). In current investigation, we found that busulfan decreased the testicular GSH levels to induce the oxidative stress. However, β-carotene not only increased testicular GSH level, but also increased the antioxidant enzymes SOD, catalase, GPX to increase testicular antioxidant capability to rescue spermatogenesis. Moreover, busulfan increased the protein level of PTEN while β-carotene decreased PTEN level to that of control which indicated that β-carotene decreased the testicular cell apoptotic level.

In summary, β-carotene rescued busulfan disrupted spermatogenesis by improving meiosis process, the expression of important genes/proteins in spermatogenesis process, testicular antioxidant capability. These beneficial advantages of β-carotene may be applied to ameliorate male reproduction for the patients who are under busulfan or other cancer-drug treatments. As natural product, β-carotene may be applied for the infertile couples especially cancer-drug treated patients by the improvement of spermatogenesis, since, worldwide many couples are infertile due to the idiopathic failed gametogenesis (spermatogenesis) (Zhou et al., 2016; Wang et al., 2018; Zhang et al., 2019).

## Data Availability

The original contributions presented in the study are included in the article/Supplementary Material, further inquiries can be directed to the corresponding authors.

## References

[B1] Aksak Karamese S. Toktay E. Unal D. Selli J. Karamese M. Malkoc I. (2015). The protective effects of beta-carotene against ischemia/reperfusion injury in rat ovarian tissue. Acta Histochem. 117 (8), 790–797. 10.1016/j.acthis.2015.07.006 26254843

[B2] Arellano-Rodriguez G. Meza-Herrera C. A. Rodriguez-Martinez R. Dionisio-Tapia R. Hallford D. M. Mellado M. (2009). Short-term intake of beta-carotene-supplemented diets enhances ovarian function and progesterone synthesis in goats. J. Anim. Physiol. Anim. Nutr. 93, 710–715. 10.1111/j.1439-0396.2008.00859.x 19141099

[B3] Boon C. S. McClements D. J. Weiss J. Decker E. A. (2010). Factors influencing the chemical stability of carotenoids in foods. Crit. Rev. Food Sci. Nutr. 50, 515–532. 10.1080/10408390802565889 20544442

[B4] Centola G. M. Blanchard A. Demick J. Li S. Eisenberg M. L. (2016). Decline in sperm count and motility in young adult men from 2003 to 2013: observations from a U.S. Sperm Bank. Andrology 4 (2), 270–276. 10.1111/andr.12149 26789272

[B5] Checa Vizcaíno M. A. Gonzalez-Comadran M. Jacquemin B. (2016). Outdoor air pollution and human infertility: a systematic review. Fertil. Steril. 106 (4), 897–904. 10.1016/j.fertnstert.2016.07.1110 27513553

[B6] Chi H. Chun K. Son H. Kim J. Kim G. Roh S. (2013). Effect of genistein administration on the recovery of spermatogenesis in the busulfan-treated rat testis. Clin. Exp. Reprod. Med. 40 (2), 60–66. 10.5653/cerm.2013.40.2.60 23875161 PMC3714430

[B7] De Bie J. Langbeen A. Verlaet A. A. J. Florizoone F. Immig I. Hermans N. (2016). The effect of a negative energy balance status on β-carotene availability in serum and follicular fluid of nonlactating dairy cows. J. Dairy Sci. 99, 5808–5819. 10.3168/jds.2016-10870 27157583

[B8] Ding N. Zhang X. Zhang X. D. Jing J. Liu S. Mu Y. (2020). Impairment of spermatogenesis and sperm motility by the high-fat diet-induced dysbiosis of gut microbes. Gut 69 (9), 1608–1619. 10.1136/gutjnl-2019-319127 31900292 PMC7456731

[B9] Dutta S. Sengupta P. (2016). Men and mice: relating their ages. Life Sci. 152, 244–248. 10.1016/j.lfs.2015.10.025 26596563

[B10] Fiedor J. Burda K. (2014). Potential role of carotenoids as antioxidants in human health and disease. Nutrients 6, 466–488. 10.3390/nu6020466 24473231 PMC3942711

[B11] Ganjalikhan Hakemi S. Sharififar F. Haghpanah T. Babaee A. Eftekhar-Vaghefi S. H. (2019). The effects of olive leaf extract on the testis, sperm quality and testicular germ cell apoptosis in male rats exposed to busulfan. Int. J. Fertil. Steril. 13 (1), 57–65. 10.22074/ijfs.2019.5520 30644246 PMC6334023

[B12] Gu L. Su Y. Zhang M. Chang C. Li J. McClements D. J. (2017). Protection of β-carotene from chemical degradation in emulsion-based delivery systems using antioxidant interfacial complexes: catechin-egg white protein conjugates. Food Res. Int. 96, 84–93. 10.1016/j.foodres.2017.03.015 28528111

[B13] Haila K. M. Nielsen B. R. Heinonen M. I. Skibsted L. H. (1997). Carotenoid reaction with free radicals in acetone and toluene at different oxygen partial pressures. Eur. Food Res. Technol. 204, 81–87. .

[B14] Han X. Zhang P. Shen W. Zhao Y. Zhang H. (2019). Estrogen receptor-related DNA and histone methylation may Be involved in the transgenerational disruption in spermatogenesis by selective toxic chemicals. Front. Pharmacol. 10, 1012. 10.3389/fphar.2019.01012 31572187 PMC6749155

[B15] Johnson E. J. (2002). The role of carotenoids in human health. Nutr. Clin. Care 5, 56–65. 10.1046/j.1523-5408.2002.00004.x 12134711

[B16] Jung S. W. Kim H. J. Lee B. H. Choi S. Kim H. Choi Y. (2015). Effects of Korean red ginseng extract on busulfan-induced dysfunction of the male reproductive system. J. Ginseng Res. 39 (3), 243–249. 10.1016/j.jgr.2015.01.002 26199556 PMC4506375

[B17] Levine H. Jørgensen N. Martino-Andrade A. Mendiola J. Weksler-Derri D. Mindlis I. (2017). Temporal trends in sperm count: a systematic review and meta-regression analysis. Hum. Reprod. Update 23 (6), 646–659. 10.1093/humupd/dmx022 28981654 PMC6455044

[B18] Li B. He X. Zhuang M. Niu B. Wu C. Mu H. (2018). Melatonin ameliorates busulfan-induced spermatogonial stem cell oxidative apoptosis in mouse testes. Antioxidants Redox Signal. 28 (5), 385–400. 10.1089/ars.2016.6792 28027652

[B19] Lin C. Choi Y. S. Park S. G. Gwon L. W. Lee G. J. Yon J. M. (2106). Enhanced protective effects of combined treatment with β-carotene and curcumin against hyperthermic spermatogenic disorders in mice. BioMed Res. Int. 2016, 2572073. 10.1155/2016/2572073 PMC5165136 28050551

[B20] Liu F. Dong W. Zhao H. Shi X. Zhang Y. (2019). Effect of molybdenum on reproductive function of male mice treated with busulfan. Theriogenology 126, 49–54. 10.1016/j.theriogenology.2018.12.002 30530157

[B21] Men Y. Zhao Y. Zhang P. Zhang H. Gao Y. Liu J. (2019). Gestational exposure to low-dose zearalenone disrupting offspring spermatogenesis might be through epigenetic modifications. Basic Clin. Pharmacol. Toxicol*.* 125 (4), 382–393. 10.1111/bcpt.13243 31058416

[B22] Merhan O. Özcan A. Atakİsİ E. ÖĞÜn M. KÜkÜrt A. (2016). The effect of β-carotene on acute phase response in diethylnitrosamine given rabbits. Kafkas Universitesi Veteriner Fakultesi Dergisi 22, 533–537. 10.9775/kvfd.2016.14995

[B23] Meza-Herrera C. A. Vargas-Beltran F. Tena-Sempere M. González-Bulnes A. Macias-Cruz U. Veliz-Deras F. G. (2013). Short-term beta-carotene-supplementation positively affects ovarian activity and serum insulin concentrations in a goat model. J. Endocrinol. Invest. 36, 185–189. 10.3275/8410 22572738

[B24] Nishino A. Yasui H. Maoka T. (2017). Reaction and scavenging mechanism of β-carotene and zeaxanthin with reactive oxygen species. J. Oleo Sci. 66, 77–84. 10.5650/jos.ess16107 27928140

[B25] Oliveira R. C. Guerreiro B. M. Morais Junior N. N. Araujo R. L. Pereira R. A. Pereira M. N. (2015). Supplementation of prepartum dairy cows with β-carotene. J. Dairy Sci. 98 (9), 6304–6314. 10.3168/jds.2014-9037 26188566

[B26] Orazizadeh M. Khorsandi L. Absalan F. Hashemitabar M. Daneshi E. (2014). Effect of beta-carotene on titanium oxide nanoparticles-induced testicular toxicity in mice. J. Assist. Reprod. Genet. 31 (5), 561–568. 10.1007/s10815-014-0184-5 24515782 PMC4016370

[B27] Pysz K. Leszczyńska T. Kopeć A. (2016). Intake of vitamin C, β-carotene, and polyphenolic compounds by children and adolescents from orphanages. J. Am. Coll. Nutr. 35, 75–85. 10.1080/07315724.2014.987405 25910044

[B28] Sies H. Stahl W. (1998). Lycopene: antioxidant and biological effects and its bioavailability in the human. Proc. Soc. Exp. Biol. Med. 218, 121–124. 10.3181/00379727-218-44285a 9605209

[B29] Skakkebaek N. E. Rajpert-De Meyts E. Buck Louis G. M. Toppari J. Andersson A. Eisenberg M. L. (2016). Male reproductive disorders and fertility trends: influences of environment and genetic susceptibility. Physiol. Rev. 96, 55–97. 10.1152/physrev.00017.2015 26582516 PMC4698396

[B30] Szczubiał M. (2015). Effect of supplementation with vitamins E, C and β-carotene on antioxidative/oxidative status parameters in sows during the postpartum period. Pol. J. Vet. Sci. 18, 299–305. 10.1515/pjvs-2015-0039 26172179

[B31] Trost L. W. Brannigan R. E. (2012). Oncofertility and the male cancer patient. Curr. Treat. Options Oncol. 13 (2), 146–160. 10.1007/s11864-012-0191-7 22528369

[B32] Vakalopoulos I. Dimou P. Anagnostou I. Zeginiadou T. (2015). Impact of cancer and cancer treatment on male fertility. Hormones (Basel) 14 (4), 579–589. 10.14310/horm.2002.1620 26732148

[B33] Virtanen H. E. Jørgensen N. Toppari J. (2017). Semen quality in the 21st century. Nat. Rev. Urol. 14, 120–130. 10.1038/nrurol.2016.261 28050014

[B34] Wang M. Liu X. Chang G. Chen Y. An G. Yan L. (2018). Single-cell RNA sequencing analysis reveals sequential cell fate transition during human spermatogenesis. Cell Stem Cell 23, 599–614. 10.1016/j.stem.2018.08.007 30174296

[B35] Wang Y. Zhao Y. Yu S. Feng Y. Zhang H. Kou X. (2016). Regulation of steroid hormones and energy status to alter spermatogenesis by cysteamine. Toxicol. Appl. Pharmacol. 313, 149–158. 10.1016/j.taap.2016.10.025 27815134

[B36] WHO (2010). WHO laboratory manual for the examination and processing of human semen. 5th Edn. Cambridge, United Kingdom: Cambridge University Press.

[B37] Yu S. Zhao Y. Feng Y. Zhang H. Li L. Shen W. (2019). β-carotene improves oocyte development and maturation under oxidative stress *in vitro* . Cell. Dev. Biol. Anim. 55 (7), 548–558. 10.1007/s11626-019-00373-0 31313007

[B38] Zhang P. Zhao Y. Zhang H. Liu J. Feng Y. Yin S. (2019). Low dose chlorothalonil impairs mouse spermatogenesis through the intertwining of estrogen receptor pathways with histone and DNA methylation. Chemosphere 230, 384–395. 10.1016/j.chemosphere.2019.05.029 31112861

[B39] Zhang W. Zhao Y. Zhang P. Hao Y. Yu S. Min L. (2018). Decrease in male mouse fertility by hydrogen sulfide and/or ammonia can be inheritable. Chemosphere 194, 147–157. 10.1016/j.chemosphere.2017.11.164 29202267

[B40] Zhao Y. Zhang P. Ge W. Feng Y. Li L. Sun Z. (2020). Alginate oligosaccharides improve germ cell development and testicular microenvironment to rescue busulfan disrupted spermatogenesis. Theranostics 10 (7), 3308–3324. 10.7150/thno.43189 32194870 PMC7053202

[B41] Zhao Y. Zhang W. Liu X. Zhang P. Hao Y. Li L. (2016). Hydrogen sulfide and/or ammonia reduces spermatozoa motility through AMPK/AKT related pathways. Sci. Rep. 6, 37884. 10.1038/srep37884 27883089 PMC5121643

[B42] Zhou Q. Wang M. Yuan Y. Wang X. Fu R. Wan H. (2016). Complete meiosis from embryonic stem cell-derived germ cells *in vitro* . Cell Stem Cell 18, 330–340. 10.1016/j.stem.2016.01.017 26923202

