# Supplementary material for: β-carotene Rescues Busulfan Disrupted Spermatogenesis Through Elevation in Testicular Antioxidant Capability
Source: Front Pharmacol. 2021 Feb 15;12:593953. doi: 10.3389/fphar.2021.593953 (PMC7917239; doi:10.3389/fphar.2021.593953)
Supplement: Supplementary file 1 [file Table1.doc]

**Table S1.** Primary antibody information

| **Gene symbol** | **Name** | **Cat. #** | **Predicted size** | **Source (Animal)** | **Company** |
| --- | --- | --- | --- | --- | --- |
| Caspase 8 | Caspas 8 | bs-0052R | 12/55kd | Rabbit (polyclonal) | Beijing Biosynthesis Biotechnology CO. |
| p-PI3K | phosphorylated Phosphoinositide 3-kinase | bs-5571R | 80kd | Rabbit (polyclonal) | Beijing Biosynthesis Biotechnology CO. |
| AKT1 | Protein kinase B | bs-0115R | 56kd | Rabbit (polyclonal) | Beijing Biosynthesis Biotechnology CO. |
| DDX4 (VASA) | DEAD (Asp Glu Ala Asp) box polypeptide | D161611 | 80kd | Rabbit (polyclonal) | Sangon Biotech (Shanghai) Co., Ltd. |
| SYCP3 | Synaptonemal complex protein 3 | D162171 | 27kd | Rabbit (polyclonal) | Sangon Biotech (Shanghai) Co., Ltd. |
| β-Actin | β-Actin | ab3280 | 42kd | Mouse(monoclonal) | Abcam |
| PTEN | Phosphatase and tensin homolog deleted on chromosome 10 (PTEN) | bs-0686R | 44kd | Rabbit (polyclonal) | Beijing Biosynthesis Biotechnology CO. |
| p-PTEN | phosphorylated PTEN | bs-3351R | 44kd | Rabbit (polyclonal) | Beijing Biosynthesis Biotechnology CO. |
| PGK2 | Phosphoglyceratekinase2 | D121803 | 45kd | Rabbit (polyclonal) | Sangon Biotech (Shanghai) Co., Ltd. |
| Piwil1 | PIWIL1 | Ab12327 | 99kd | Rabbit (polyclonal) | Abcam |
| SOD | Superoxide dismutase 1 | bs-1080R | 22kd | Rabbit (polyclonal) | Beijing Biosynthesis Biotechnology CO |
| P53 | Tumor protein p53 | bs-8687R | 53kd | Rabbit (polyclonal) | Beijing Biosynthesis Biotechnology CO |
| AR | Androgen receptor | bs-0118R | 43/101kd | Rabbit (polyclonal) | Beijing Biosynthesis Biotechnology CO |
