# Supplementary figures and images for: β-carotene Rescues Busulfan Disrupted Spermatogenesis Through Elevation in Testicular Antioxidant Capability
Source: Front Pharmacol. 2021 Feb 15;12:593953. doi: 10.3389/fphar.2021.593953 (PMC7917239; doi:10.3389/fphar.2021.593953)

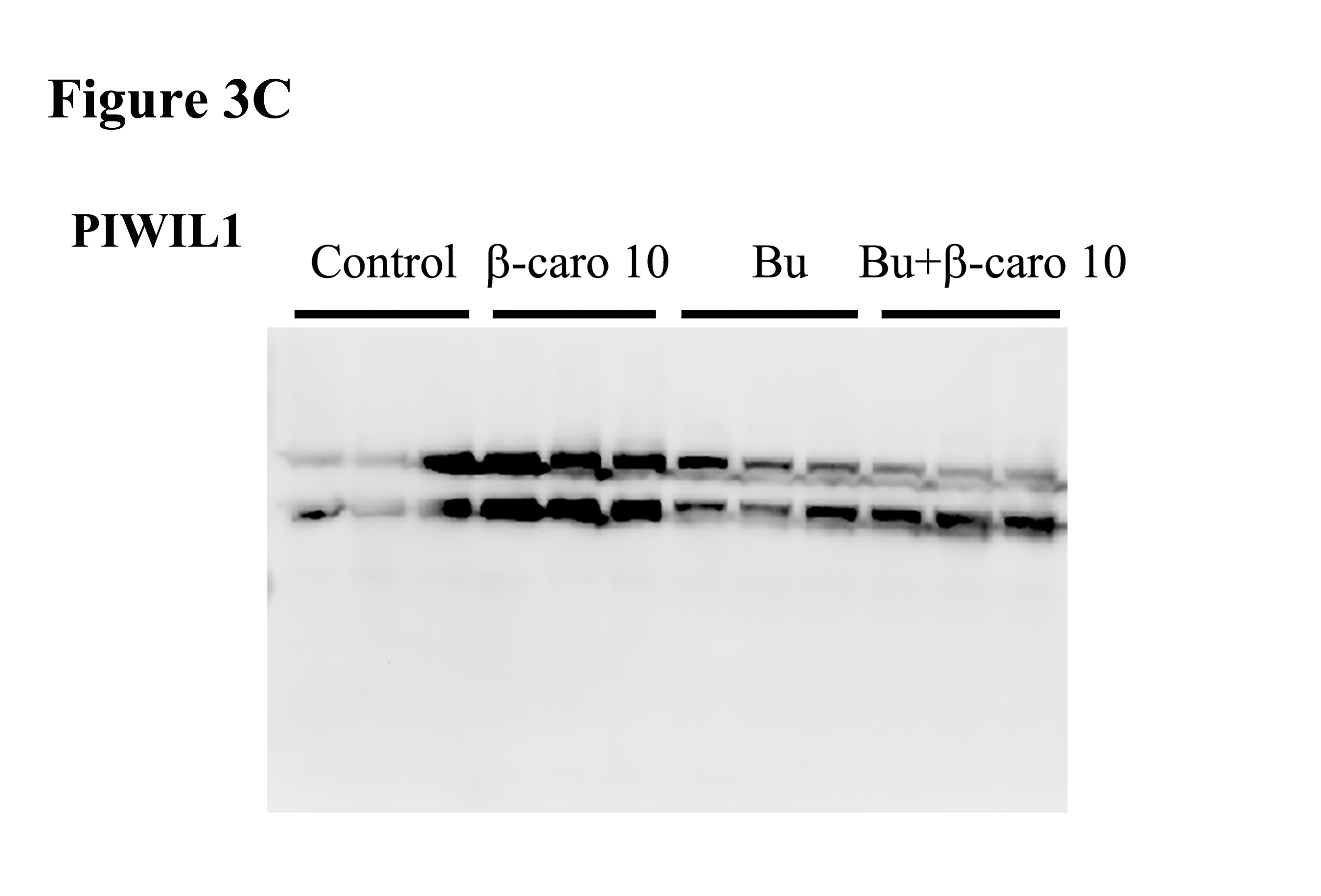

Supplement: Supplementary file 2 [file Image1.tif]

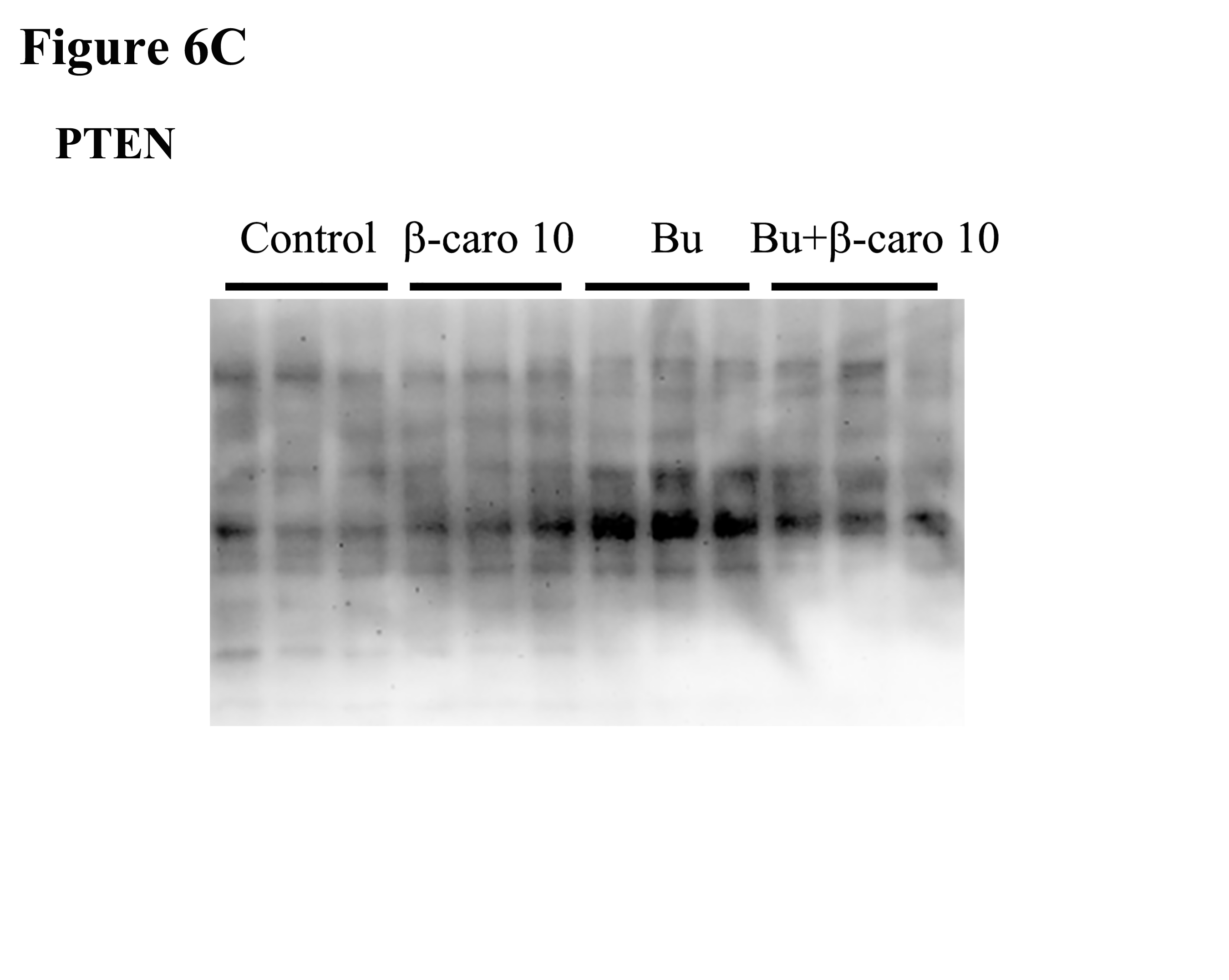

Supplement: Supplementary file 3 [file Image2.tif]

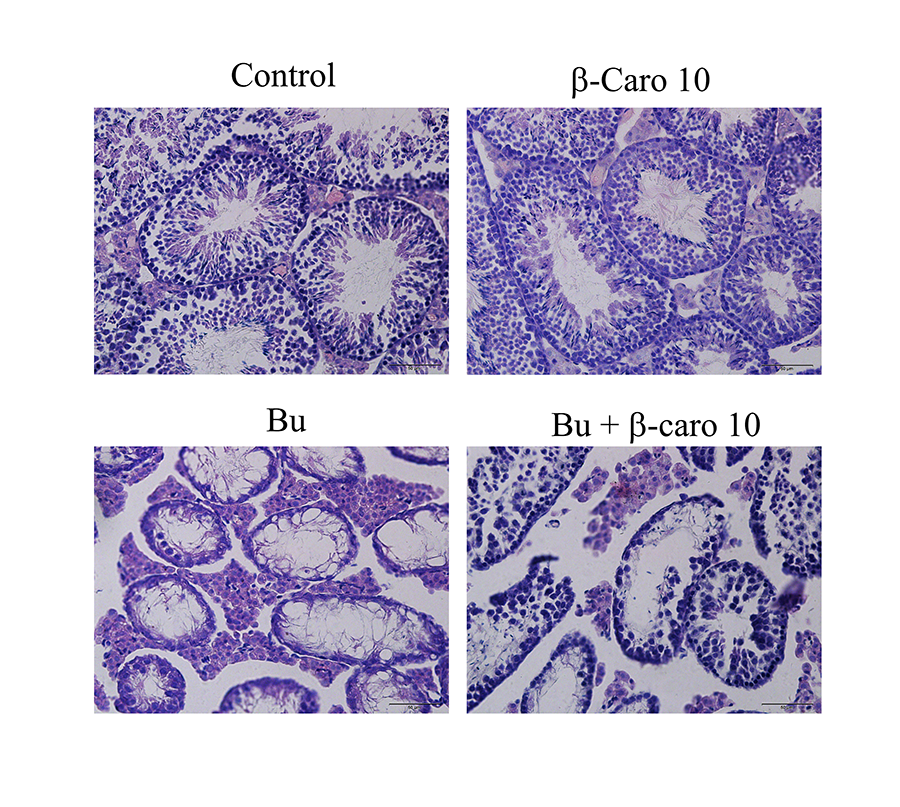

Supplement: Supplementary file 4 [file Image3.tif]
