# Supplementary material for: β-carotene Rescues Busulfan Disrupted Spermatogenesis Through Elevation in Testicular Antioxidant Capability
Source: Front Pharmacol. 2021 Feb 15;12:593953. doi: 10.3389/fphar.2021.593953 (PMC7917239; doi:10.3389/fphar.2021.593953)
Supplement: Supplementary file 5 [file DataSheet1.docx]

**Supplemental Information**

**Detailed Materials and Methods**

***Evaluation of spermatozoa motility using a computer-assisted sperm analysis system***

Spermatozoa motility was assessed using a computer-assisted sperm assay (CASA) method according to World Health Organization guidelines (Zhang et al., 2018; Zhao et al., 2020). After euthanasia, spermatozoa were collected from the cauda epididymis of mice and suspended in DMEM/F12 medium with 10% FBS and incubated at 37.5 ℃ for 30 min; samples were then placed in a pre-warmed counting chamber (Zhang et al., 2018; Zhao et al., 2020). A microscopic sperm class analyzer (CASA; MICROPTIC S.L. Barcelona, Spain) was used in this investigation. It was equipped with a 20-fold objective, a camera adaptor (Eclipse E200, Nikon, Japan), and a camera (acA780-75gc, Basler, Germany). The classification of sperm motility was as follows: Grade A linear velocity >22 μm s^-1^; Grade B <22 μm s^-1^ and curvilinear velocity >5 μm s^-1^; Grade C curvilinear velocity <5 μm s^-1^; and Grade D = immotile spermatozoa. The spermatozoa motility data represented only Grade A + Grade B since only these two grades are considered to be functional.

**Morphological observations of spermatozoa**

The extracted murine caudal epididymides were placed in RPMI medium and finely chopped; subsequently, Eosin Y (1%) was added for staining as described previously (Zhang et al., 2018; Zhao et al., 2020). Spermatozoon abnormalities were then viewed using an optical microscope and were classified into head or tail morphological abnormalities: two heads, two tails, blunt hooks, and short tails. The examinations were repeated three times, and 500 spermatozoa per animal were scored.

***Assessment of acrosome integrity***

After harvest, mouse spermatozoa were incubated at 37.5 ℃ for 30 min, after which a drop of sperm suspension was uniformly smeared on a clean glass slide. Smeared slides were air dried and incubated in methanol for 2 min for fixation. After fixation, the slides were washed with PBS three times. Assessment of an intact acrosome was accomplished by staining the sperm with 0.025% Coomassie brilliant blue G-250 in 40% methanol for 20 min at room temperature (RT). The slides were then washed three times with PBS and mounted with 50% glycerol in PBS. Acrosomal integrity was determined by an intense staining on the anterior region of the sperm head under bright-field microscopy (AH3-RFCA, Olympus, Tokyo, Japan) and scored accordingly (Zhang et al., 2018; Zhao et al., 2020).

***RNA-seq analysis***

Transcriptomics were analyzed as described in our earlier article (Zhao et al., 2016). Briefly, total RNA was isolated using TRIzol Reagent (Invitrogen) and purified using a Pure-Link1 RNA Mini Kit (Cat: 12183018A; Life Technologies) following the manufacturer’s protocol. The total RNA samples were first treated with DNase I to degrade any possible DNA contamination. Then the mRNA was enriched using oligo(dT) magnetic beads. When mixed with the fragmentation buffer, the mRNA was broken into short fragments (about 200 bp), after which the first strand of cDNA was synthesized using a random hexamer-primer. Buffer, dNTPs, RNase H, and DNA polymerase I were added to synthesize the second strand. The double strand cDNA was purified with magnetic beads. Subsequently, 3'-end single nucleotide A (adenine) addition was performed. Finally, sequencing adaptors were ligated to the fragments. The fragments were enriched by PCR amplification. During the QC step, Agilent 2100 Bioanalyzer and ABI StepOnePlus Real-Time PCR System were used to qualify and quantify the sample library. The library products were ready for sequencing via Illumina HiSeqTM 2500. The reads were mapped to reference genes using SOAPaligner (v. 2.20) with a maximum of two nucleotide mismatches allowed at the parameters of “-m 0 -x 1000 -s 40 -l 35 -v 3 -r 2”. The read number of each gene was transformed into RPKM (Reads Per Kilo bases per Million reads), and then differentially expressed genes were identified using the DEGseq package and the MARS (MA-plot-based method with the Random Sampling model) method. The threshold was set as FDR ≤0.001 and an absolute value of log_2_ ratio ≥1 to judge the significance of differences in gene expression.

***Histopathological analysis***

Testicular tissues were fixed in 10% neutral buffered formalin, paraffin embedded, cut into 5 μm sections and subsequently stained with hematoxylin and eosin for histopathological analysis.

***Immunofluorescence staining (IHF)***

The procedure for immunofluorescence staining is reported in our recent publications (Zhang et al., 2018; Zhao et al., 2020). Table S8 lists the primary antibodies. Briefly, testis sections (5 μm) were prepared and subjected to antigen retrieval. Sections were then blocked with normal goat serum in PBS, followed by incubation (1:150 in PBS-1% BSA) with primary antibodies at 4 ^o^C overnight. After a brief wash, sections were incubated with goat anti-rabbit or donkey anti-goat secondary Abs (1:100 in PBS; Beyotime Institute of Biotechnology, Shanghai, P.R. China) at RT for 30 min and then counterstained with 4',6-diamidino-2-phenylindole (DAPI). The stained sections were visualized using a Nikon Eclipse TE2000-U fluorescence microscope (Nikon, Inc., Melville, NY), and the captured fluorescence images were analyzed using MetaMorph software. Six samples/group were determined.

***Western blotting***

Western blotting analysis followed the procedure reported in our previous publications (Zhang et al., 2018; Zhao et al., 2020). Briefly, testis tissue samples were lysed in RIPA buffer containing a protease inhibitor cocktail from Sangong Biotech, Ltd. (Shanghai, China). Protein concentration was determined by BCA kit (Beyotime Institute of Biotechnology). Information for primary antibodies is given in Table S8. Secondary donkey anti-goat Ab (Cat no.: A0181) was purchased from Beyotime Institute of Biotechnology, and goat anti-rabbit (Cat no.: A24531) Abs were purchased from Novex^®^ by Life Technologies (USA). Fifty micrograms of total protein per sample were loaded onto 10% SDS polyacrylamide electrophoresis gels. The gels were transferred to a polyvinylidene fluoride (PVDF) membrane at 300 mA for 2.5 h, at 4 ℃. Then, the membranes were blocked with 5% BSA for 1 h at RT, followed by three washes with 0.1% Tween-20 in TBS (TBST). The membranes were incubated with primary Abs diluted with 1:500 in TBST with 1% BSA overnight at 4 ℃. After three washes with TBST, the blots were incubated with the HRP-labelled secondary goat anti-rabbit or donkey anti-goat Abs respectively for 1 h at RT. After three washes, the blots were imaged. The experiment was performed with six individual samples/group.

***Statistical analysis***

Data were analyzed using SPSS statistical software (IBM Co., NY, USA) with one-way analysis of variance (ANOVA) followed by LSD multiple comparison tests. All groups were compared with each other for every parameter. The data were shown as the mean ± SEM. Statistical significance was based on *p* < 0.05.
